# Capturing the domain crosstalk in full length LRRK2 and LRRK2^RCKW^

**DOI:** 10.1042/BCJ20230126

**Published:** 2023-06-12

**Authors:** Eliza Störmer, Jui-Hung Weng, Jian Wu, Daniela Bertinetti, Pallavi Kaila Sharma, Wen Ma, Friedrich W. Herberg, Susan S. Taylor

**Affiliations:** 1 Department of Biochemistry, University of Kassel, Kassel, Germany; 2 Department of Pharmacology, University of California, San Diego, U.S.A.; 3 Department of Chemistry and Biochemistry, University of California, San Diego, U.S.A.

**Keywords:** GTPases, kinases, leucine rich repeat kinase, molecular dynamics, Parkinsons disease

## Abstract

LRR-kinase 2 (LRRK2) is a multi-domain protein with three catalytically inert N-terminal domains (NtDs) and four C-terminal domains, including a kinase and a GTPase domain. LRRK2 mutations are linked to Parkinson's Disease (PD). Recent structures of LRRK2^RCKW^ and a full-length inactive LRRK2 (fl-LRRK2^INACT^) monomer revealed that the kinase domain drives LRRK2 activation. The LRR domain and also an ordered LRR–COR linker, wrap around the C-lobe of the kinase domain and sterically block the substrate binding surface in fl-LRRK2^INACT^. Here, we focus on the crosstalk between domains. Our biochemical studies of GTPase and kinase activities of fl-LRRK2 and LRRK2^RCKW^ reveal how mutations influence this crosstalk differently depending on the domain borders investigated. Furthermore, we demonstrate that removing the NtDs leads to altered intramolecular regulation. To further investigate the crosstalk, we used Hydrogen–Deuterium exchange Mass Spectrometry (HDX-MS) to characterize the conformation of LRRK2^RCKW^ and Gaussian Accelerated Molecular Dynamics (GaMD) to create dynamic portraits of fl-LRRK2 and LRRK2^RCKW^. These models allowed us to investigate the dynamic changes in wild-type and mutant LRRK2s. Our data show that the α3^ROC^ helix, the Switch II motif in the ROC domain, and the LRR–ROC linker play crucial roles in mediating local and global conformational changes. We demonstrate how these regions are affected by other domains in fl-LRRK2 and LRRK2^RCKW^ and show how unleashing of the NtDs as well as PD mutations lead to changes in conformation and dynamics of the ROC and kinase domains which ultimately impact kinase and GTPase activities. These allosteric sites are potential therapeutic targets.

## Introduction

**Figure 1.**
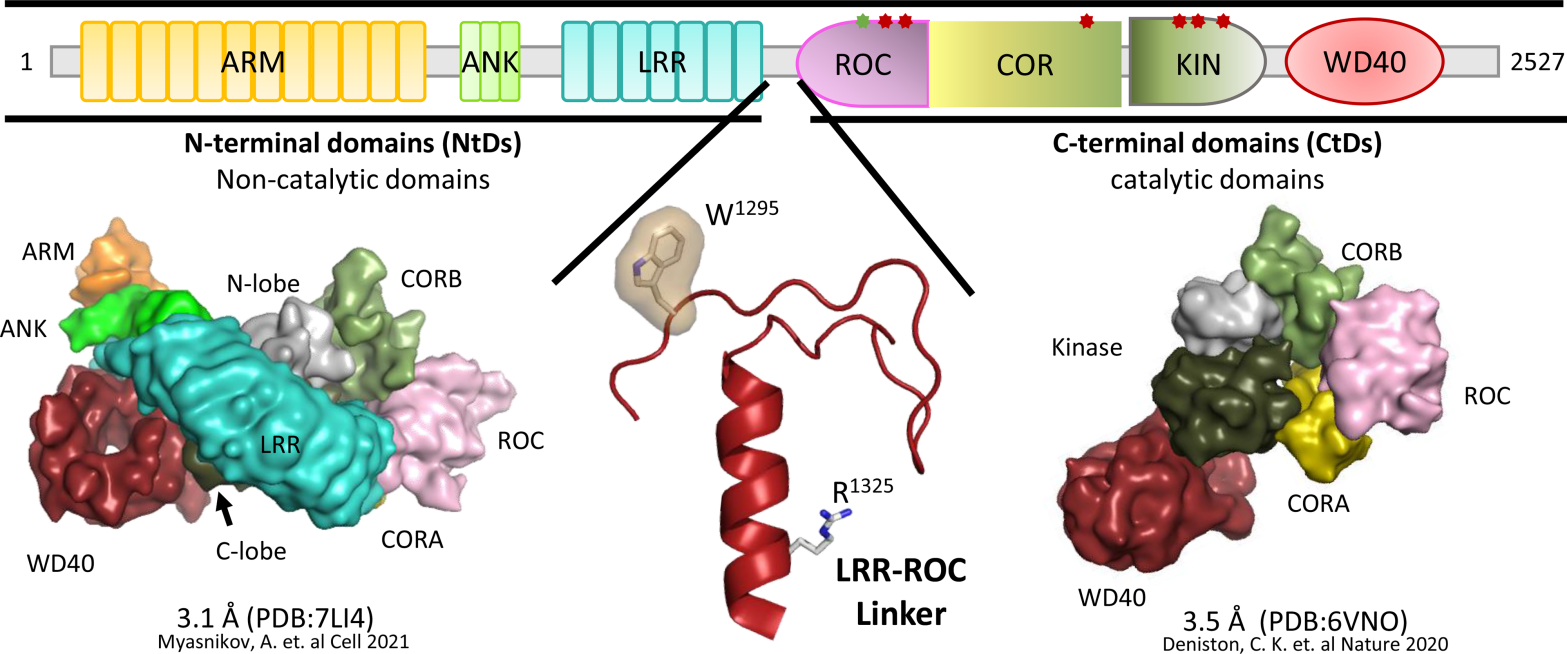
Domain organization of LRRK2 and structures of fl-LRRK2 and LRRK2^RCKW^. The domain organization of LRRK2 is summarized at the top, including key Parkinson Disease Mutations. In addition to the seven well-folded domains in full-length inactive LRRK2 there is an additional stable motif that links the LRR domain and the ROC domain. This motif, LRR–ROC Linker (bottom, middle), is disordered in active fl-LRRK2 and missing in LRRK2^RCKW^. Two key residues in this motif are W1295 described previously (JMB) and R1325 which was recently described as a PD mutation site [3,20]. Here we explore the domain dynamics in two cryo-EM structures. On the bottom left is inactive fl-LRRK2^INACT^ (pdb: 7li4); on the right bottom is LRRK2^RCKW^ (pdb: 6vno). GaMD simulations create a dynamic portrait of each static cryo-EM structure.

**Figure 2.**
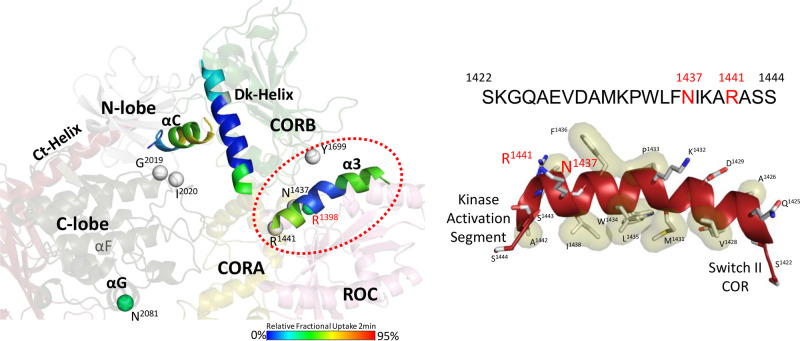
Helical Motifs in LRRK2^RCKW^ drive the crosstalk between domains in LRRK2. The helical motifs in the CORB domain and the C-terminal (Ct) helix flank the kinase domain while the α3^ROC^ Helix in the ROC domain dominates the interface between the ROC and CORB domains, where many PD mutations are localized (red circle). The solvent exposure of each helix is illustrated by color coding based on HDX-MS data. (Left) The α3^ROC^ helix is very hydrophobic and creates an interface with the CORB domain on one surface and is docked up against Switch II on the other surface (Right).

Several strategically positioned mutations cause the large multi-domain intracellular LRR-kinase 2 (LRRK2) to become a risk factor for Parkinson's Disease (PD). LRRK2 contains seven well-folded domains joined by a linker motif that is well-ordered in full-length inactive LRRK2 (fl-LRRK2^INACT^) but becomes disordered in active fl-LRRK2 where it now becomes a highly dynamic motif that links the three catalytically inert N-terminal domains (NtDs) and the C-terminal domains (CtDs) [1–3] (Figure 1). The four CtDs contain two catalytic domains, a kinase domain and a ROC (Ras-of-complex) domain, which is a GTPase. While kinases and GTPases, two of the most important biological switches in biology, communicate with each other in many signaling pathways, LRRK2 is one of the few examples where both a kinase and a GTPase domain are embedded in the same polypeptide chain. Since its discovery as a risk factor for PD in 2004 [4,5], much has been learned about the activation and the biological targets of LRRK2. In terms of regulation, the conformational changes associated with activation of LRRK2 correlate with activation of the kinase domain, and two of the most important mutations (G2019S and I2020T) that drive activation, are located in the critical DFG motif (DYG in LRRK2). Activating mutations in the DFG motif are common in other kinases; this motif is actually a ‘hot spot' for tumor-driving mutations in protein kinases as well as for this neurodegenerative phenotype [6]. This motif drives the switch that leads to opening and closing of the catalytic cleft which defines the interface between the N-lobe and the C-Lobe of the kinase domain. The other ‘hot spot' for PD mutations in LRRK2 is at the ROC:CORB interface (Figure 2). In contrast with the DFG motif, which is highly conserved in all kinases, the ROC:CORB interface is unique to LRRK2 and the ROCO proteins [7,8]. Many PD mutations cluster at this interface, and these residues are not highly conserved. Here we explore the protein:protein interfaces that bridge both catalytic cores as well as the motifs that are embedded in the ROC domain.

Until recently there has been a paucity of information regarding LRRK2 structure, most likely because the kinase domain cannot be expressed as a stable active protein on its own. Removing even a few residues from the C-terminus leads to inactivation [9]. Based on cryo-Electron Microscopy (cryo-EM) and cryo-Electron Tomography (cryo-ET), as well as classic X-ray crystallography, coupled with biochemistry and cell biology functional assays, two multi-domain structures were published in 2020 following the structure of the C-terminal WD40 domain in 2019 [10]. The four CtDs were included in the high resolution (3.5 Å) cryo-EM structure of a LRRK2 monomer [8], LRRK2^RCKW^, while a lower resolution (13 Å) structure of a polymerized full-length active LRRK2 dimer wrapped in a helical array around microtubules was solved by cryo-ET [11]. A high resolution (3.1 Å) cryo-EM structure of a full-length inactive monomer followed shortly [1]. The most recent cryo-EM structure of full-length active LRRK2 demonstrates the remarkable flexibility of LRKK2 as well as the critical importance of the oligomeric states of LRRK2 [2]. As predicted previously, it is the kinase domain that drives the conformational dynamics in LRRK2^RCKW^, and the kinase domain is the only domain that touches all of the other CtDs except the ROC domain which is close proximity to the C-lobe of the kinase domain [12]. In fl-LRRK2^INACT^, the linker interacts with the C-lobe of the kinase domain, the ROC:CORA domains, and the LRR domain, and stabilize the closed conformation that locks the LRR domain over the active site of the kinase domain [3].

Using Hydrogen Deuterium exchange coupled with Mass Spectrometry (HDX-MS) we were able to observe how the loops and linkers are more solvent exposed versus regions that were shielded from solvent due to their secondary structure (Figure 2). By coupling HDX-MS with Gaussian accelerated Molecular Dynamic (GaMD) simulations, we then created a dynamic portrait of the static cryo-EM structure of LRRK2^RCKW^ (pdb: 6vno) [8,13]. The cryo-EM structure of fl-LRRK2^INACT^ (pdb: 7li4) shows how the NtDs [1], in particular the LRR domain, wrap around the C-lobe of the kinase domain and sterically block the substrate binding surface, in particular the P + 1 binding pocket [3]. This full-length structure also includes a novel stable motif that links the LRR domain to the ROC domains (Figure 1). Using the same strategy, we are now using GaMD simulations coupled with biochemical and kinetic data to decipher the crosstalk between the kinase domain and the GTPase/ROC domain with the goal of elucidating how PD mutations at the ROC:CORB interface and at the Linker:ROC:CORA interface alter the domain dynamics that mediate this crosstalk.

In our earlier analyses, we highlighted the importance of the CORB Docking helix (Dk-helix) that is anchored to the αC helix in the N-Lobe of the kinase domain, and the C-terminal helix (Ct-helix) that is stably anchored to the C-Lobe of the kinase domain [13]. Here we focus on the α3 helix in the ROC domain (α3^ROC^), which not only creates the interface between the ROC and CORB domains but also mediates crosstalk with Switch II in the ROC domain and the Activation Segment in the kinase domain (Figure 2). In addition, several PD mutations are at this interface. Using GaMD and HDX-MS, we begin to define more rigorously the biophysical and dynamic features of the ROC domain and then show how PD mutations, and other mutations, influence crosstalk between the ROC domain and the kinase domain contrasting fl-LRRK2^INACT^ and LRRK2^RCKW^. How does the ROC domain receive and transmit information to the other domains of LRRK2? How is it silenced in fl-LRRK2^INACT^ and prevented from communicating to the kinase domain, and how is its ability to communicate unleashed when LRRK2 is activated? How is the network altered when the NtDs are removed leaving only the catalytic CtDs? Finally, how are the function and dynamics of fl-LRRK2^INACT^ and LRRK2^RCKW^ altered by PD mutations? Our results capture global differences in both biochemistry and dynamics between fl-LRRK2 and LRRK2^RCKW^ and highlight the importance of the α3^ROC^ helix, Switch II and the LRR–ROC linker in mediating local and global changes.

## Results

### Biochemical and functional analysis of fl-LRRK2 and LRRK2^RCKW^

**Figure 3.**
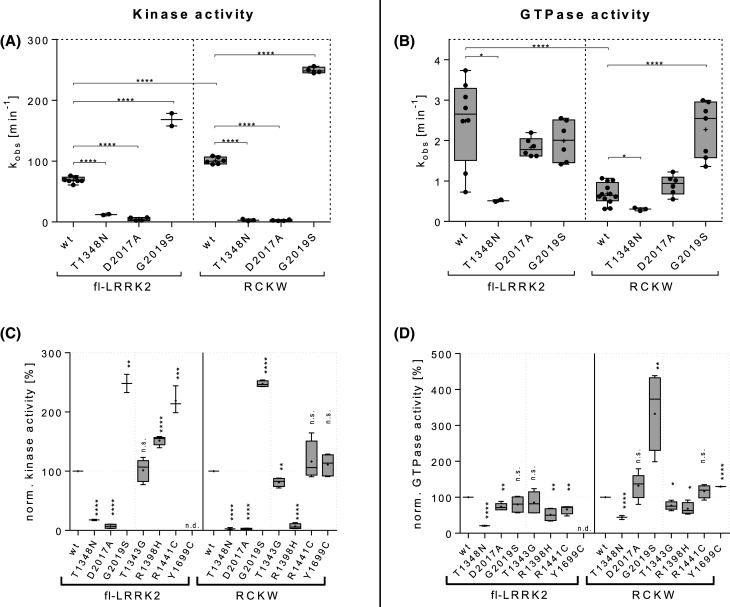
Biochemical and functional analysis of fl-LRRK2 and LRRK2^RCKW^. (**a**) Absolute kinase turnover numbers k_obs_ [min^−1^], determined with MMSA, show generally higher activity in LRRK2^RCKW^ than in fl-LRRK2 regarding the wild type and the PD pathogenic G2019S mutation. Both control mutations (GTP binding deficient T1348N and kinase dead D2017A) show negligible kinase activity in both constructs. Significances were tested with parametric *t*-test (two-sided, confidence interval 95%) **** *P* < 0.0001, *** *P* ≤ 0.001, ** *P* ≤ 0.01, * *P* ≤ 0.05, n.s.: not significant. (**b**) Absolute GTPase turnover numbers k_obs_ [min^−1^], determined with the radioactive charcoal assay, show generally higher activity in fl-LRRK2 than in LRRK2^RCKW^ comparing both wild types and D2017A mutation which shows no effect on neither of the constructs. T1348N decreases GTP turnover in both constructs and G2019S increases activity in LRRK2^RCKW^ to fl-LRRK2 levels while leaving activity unaffected in fl-LRRK2. Significances were tested with parametric *t*-test (two-sided, confidence interval 95%) **** *P* < 0.0001, *** *P* ≤ 0.001, ** *P* ≤ 0.01, * *P* ≤ 0.05, n.s.: not significant. (**c** and **d**) Enzymatic activities normalized to respective wild-type levels display differences in the respective effects of mutations on fl-LRRK2 and LRRK2^RCKW^. Most apparent are the PD related mutations R1398H, R1441C and G2019S which each increase either kinase activity (R1398H and R1441C) or GTPase activity (G2019S) in one construct while leaving the respective activity mainly unaffected (R1441C and G2019S) or decreasing it (R1398H) in the other construct (n.d.: not determined).Significances were tested with parametric *t*-test (two-sided, confidence interval 95%) **** *P* < 0.0001, *** *P* ≤ 0.001, ** *P* ≤ 0.01, * *P* ≤ 0.05, n.s.: not significant. A complete list of all absolute and percentage activities is shown in Supplementary Figure S1.

Comparing the respective wild-type proteins, LRRK2^RCKW^ shows a 1.5-fold increase in kinase activity compared with fl-LRRK2 while its GTPase is less than a third as active as in fl-LRRK2 (Figure 3A,B). The PD pathogenic mutation G2019S increases kinase activity in both constructs by about the same factor (Figure 3C) whereas, it does not significantly affect GTPase activity in fl-LRRK2 but increases it in LRRK2^RCKW^ to the same level as fl-LRRK2 (Figure 3B). The reported GTP binding deficient mutation T1348N shows drastically reduced GTPase activity as expected but also negligible kinase activity [14–16]. Thus, it cannot be confirmed if this mutation results in a functional LRRK2 protein. In contrast, the kinase dead D2017A mutation shows unaffected GTP hydrolysis in fl-LRRK2 as well as in LRRK2^RCKW^ while it reduces kinase activity to negligible levels in both constructs (Figure 3A,B).

Besides the pathogenic G2019S mutation, which is located in the kinase domain, three more PD mutations showed different effects on both constructs. R1441C, located in the ROC domain does not affect kinase or GTPase activity significantly in LRRK2^RCKW^, while it strongly increases kinase activity and slightly decreases GTPase activity in fl-LRRK2 compared with wt fl-LRRK2 (Figure 3C,D). Y1699C, which is located in the CORB domain, shows no effect on kinase activity but slightly enhanced GTPase activity in LRRK2^RCKW^ compared with wt LRRK2^RCKW^ (Figure 3D). Unfortunately, we were not able to produce fl-LRRK2 Y1699C, but it is reported to show reduced GTP hydrolysis [17], whereas its reported effect on kinase activity seems to depend on the experimental setup and substrate. Surprisingly, the protective R1398H mutation, that is located in the Switch II motif of the ROC domain, appears to decrease kinase activity in LRRK2^RCKW^ almost as potently as the kinase dead D2017A mutation, whereas it elevates kinase activity in fl-LRRK2 and GTPase activity are lowered in both constructs, compared with the respective wild types (Figure 3C,D). Hence, the three PD related mutations (R1398H, R1441C and G2019S) affect the two enzymatic activities in very different ways when we compare fl-LRRK2 with LRRK2^RCKW^ (Supplementary Figure S1, red rectangles), suggesting that crosstalk between the NtDs and CtDs is an important part of the signaling mechanism.

An interesting feature that the cryo-EM structure of LRRK2^RCKW^ revealed is a phosphoryl group at T1343 within the ROC domain that was not present in the cryo-EM structure of fl-LRRK2^INACT^. To address the question, of whether the phosphorylation is critical for enzymatic activity, we mutated this site to glycine (corresponding to G12 in Ras) and quantified both activities. The T1343G substitution has no significant effect on fl-LRRK2 and slightly decreases both kinase and GTPase activities in LRRK2^RCKW^ compared with wt LRRK2^RCKW^ (Figure 3C,D). These differences, found by comparing both enzymatic activities of fl-LRRK2 and LRRK2^RCKW^, suggest that removing the NtDs leads to altered intramolecular regulation. We thus compared both cryo-EM structures *in situ*.

### Capturing domain dynamics with Gaussian accelerated MD simulations

**Figure 4.**
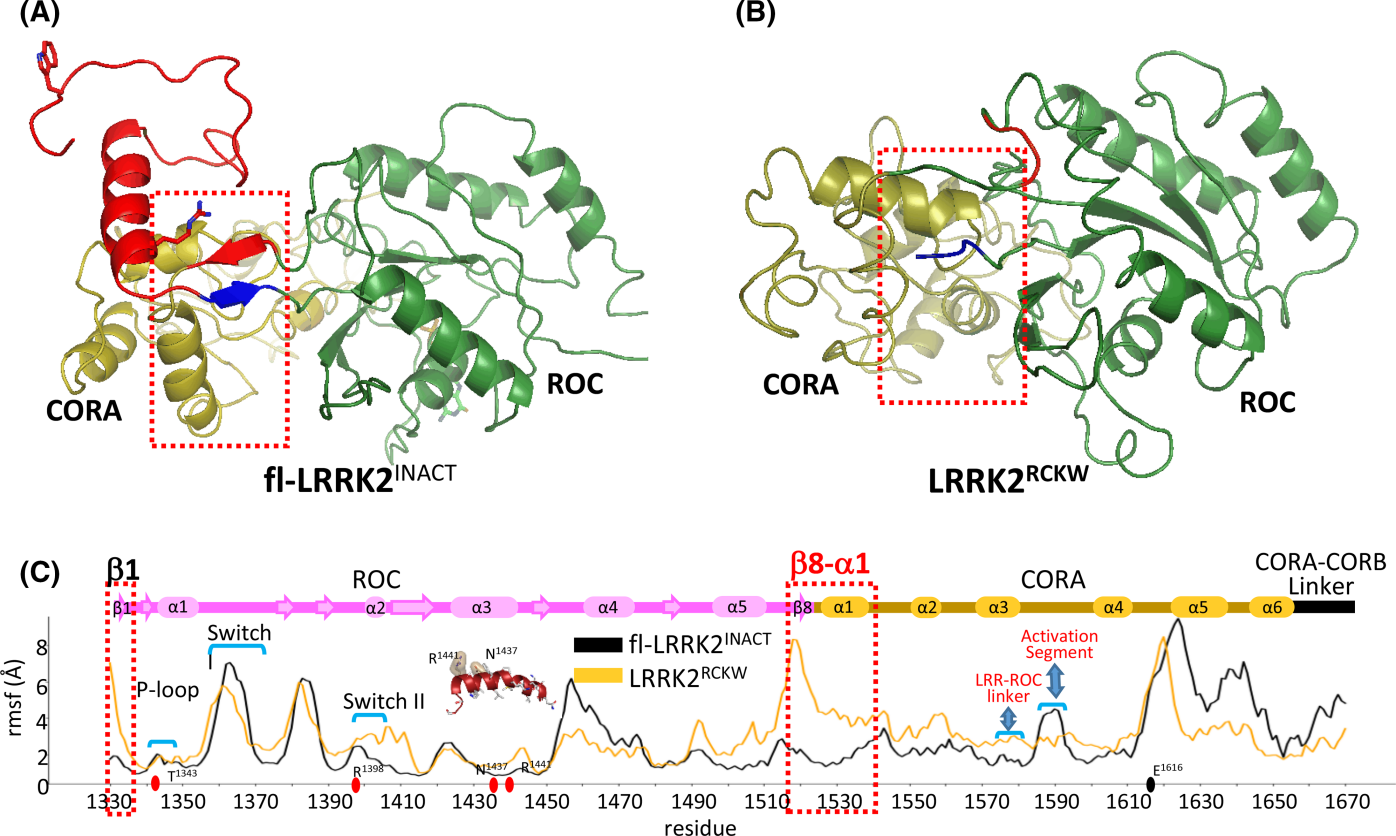
Gaussian MD Simulations of the ROC:COR domains in fl-LRRK2 and LRRK2^RCKW^. The GaMD representative structures of fl-LRRK2^INACT^ (top left) and LRRK2^RCKW^ (top right) are shown. Only fl-LRRK2^INACT^ includes the LRR–ROC linker motif, colored in red. The ROC and COR-A domains are colored in green and gold, respectively. In the RMSF analysis (bottom), LRRK2^RCKW^ shows higher RMSF for β1 and β8 of ROC domain and the first helix in CORA domain compared with fl-LRRK2^INACT^. The figure highlights switch I, switch II, P-loop, and α3^ROC^ helix in the ROC domain, as well as regions in the CORA domain that interact with the LRR–COR linker and the Activation Segment.

To compare the dynamics of the CtDs with and without the NtDs, we used GaMD simulations to create the dynamic portrait of fl-LRRK2 and LRRK2^RCKW^. The simulations were based on two reported cryo-EM structures: (1) LRRK2^RCKW^ (pdb: 6vno) that contains only the four C-terminal domains (CtDs) and includes both catalytic domains; and (2) fl-LRRK2^INACT^ (pdb:7lhw), which includes parts of the ARM and the entire ANK, LRR and the CtDs (Figure 1). As seen in Figure 4A,B, the global dynamics of the ROC:COR domains are dramatically different. Based on the RMSF analysis of each residue, we can identify the dynamic differences in particular regions (Figure 4C). For example, we can see that the β1/β8 strands that begin and end the ROC domain and portions of Switch II (residues 1395–1409) are more stable in fl-LRRK2^INACT^, while in LRRK2^RCKW^, the other regions appear to be more stable. To understand these differences, we needed to first define the motifs of the ROC domain. Based on the GaMD simulations and the HDX-MS data, which was described previously [13], we wanted to understand if the HDX-MS data capture the difference in dynamics that are revealed by the GaMD simulations. Most importantly, we wanted to know how the dynamics correlate with the altered position of R1398 in the two structures. R1398H is a protective mutation in both PD and Inflammatory bowel disease (IBD) [18,19]. Can the differences in dynamics explain why the kinase activity in fl-LRRK2 is increased when R1398 is mutated to His? We will first explore two motifs in the ROC domain, the α3^ROC^ helix and Switch II, and then return to the altered dynamics in fl-LRRK2^INACT^ vs. LRRK2^RCKW^.

### Motifs in the ROC domain

**Figure 5.**
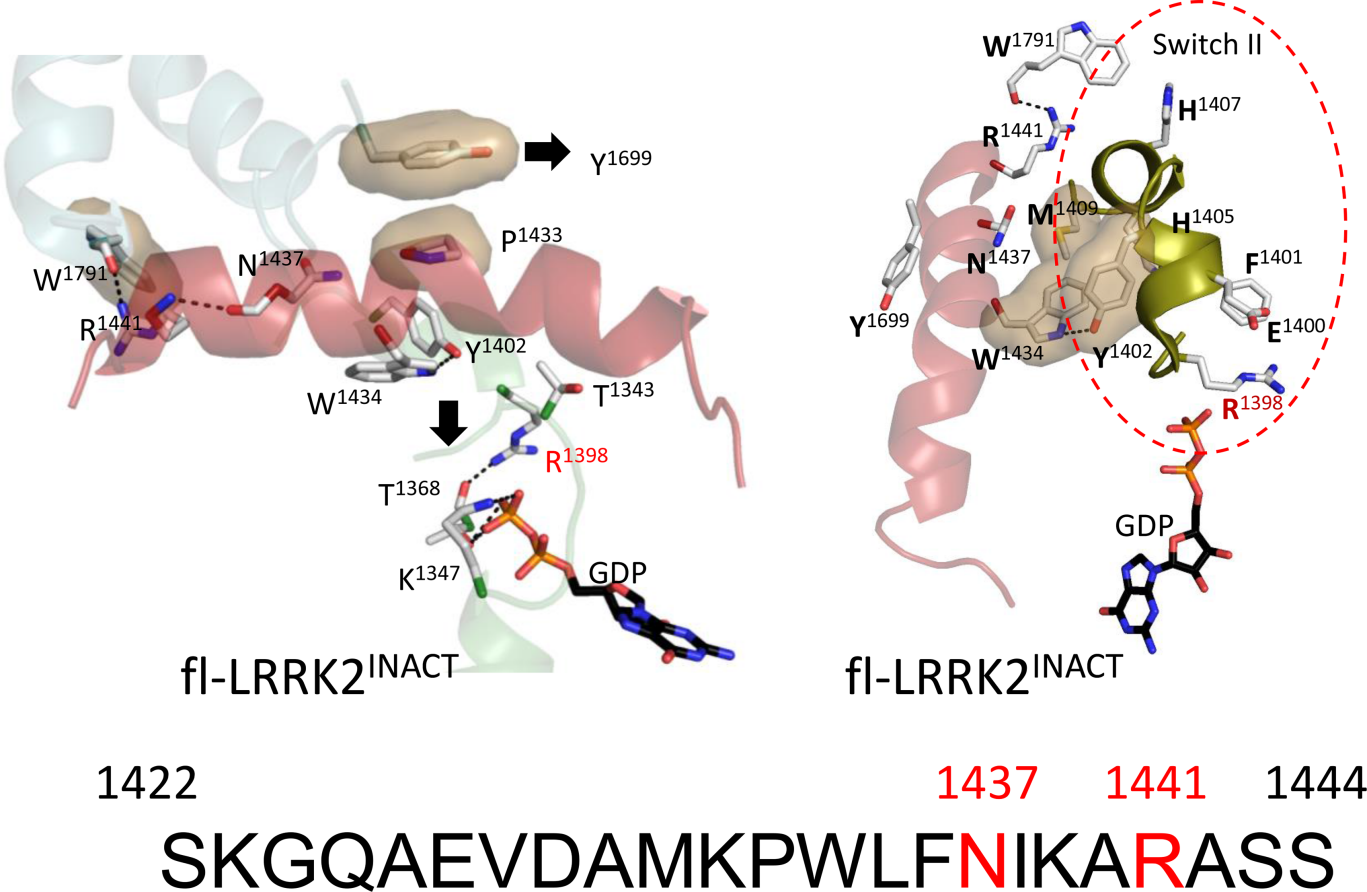
α3^ROC^ Helix in the fl-LRRK2. The left panel shows the α3^ROC^ Helix of fl-LRRK2^INACT^ with highlighted key interactions, such as R1441 at the C-terminal end interacting with W1791 on the C-terminal end of the Dk-helix, R1398 pointing down interacting with T1368, and K1347 interacting with the GDP phosphate group. In this inactive conformation, residue Y1699 in the COR-B domain is pointing towards the N-terminus of the α3^ROC^ Helix. The right panel highlights key residues in the switch II region of the ROC domain (red circle). The sequence of the α3^ROC^ Helix is shown at the bottom, with PD mutation sites highlighted in red.

Our previous analysis of the domains and motifs of LRRK2 identified two dominant helices that flank the kinase domain, the Docking helix (Dk-helix) in CORB that is anchored to the αC helix of the kinase domain and the C-terminal helix (Ct-helix) that follows the WD40 domain and docks onto the C-lobe of the kinase domain [12,13]. Within the kinase domain there are two functionally dominant helices — the hydrophobic αF helix that is buried within the C-lobe and the critical αC helix in the N-lobe that in LRRK2 is anchored firmly to the CORB Dk-helix [13]. The dominant feature of the ROC domain is the α3^ROC^ helix that creates an interface with the CORB domain on one surface and is docked up against Switch II on the other surface (Figures 2 and 5). The α3^ROC^ helix also harbors several PD mutations**.**

### α3^ROC^ helix

The most striking feature of the α3^ROC^ helix is its hydrophobicity (Figure 2). The entire central core of this helix is very hydrophobic and completely shielded from solvent based on HDX-MS [13]. One side of the hydrophobic surface is anchored firmly to the core of the ROC domain while the other hydrophobic surface is anchored to the COR-B domain. In terms of its hydrophobicity the α3^ROC^ helix is like the αF helix of the kinase domain. Surprisingly neither of these helices are predicted by standard secondary structure predictors, in part because both have a helix breaker residue in the middle. In the case of the αF helix it is G2060 that breaks the helix, whereas in the α3^ROC^ helix it is P1433. Both helices are thus dependent on the surrounding environment and are slightly ‘kinked' in the middle; neither would form spontaneously.

Y1699, located at the N-terminus of a short dynamic helix in CORB (Figure 5 and Supplementary Figure S2), is a critical contact point between the ROC domain and the CORB domain. In fl-LRRK2^INACT^ it docks onto P1433 in the α3^ROC^ helix. This position of Y1699, which is also a PD mutation site, is predicted to be a marker for the inactive versus the active conformation of LRRK2 [2]. In fl-LRRK2^INACT^ Y1699 is in an ‘inactive' conformation. While the middle segment of the α3^ROC^ helix, like the αF helix in the kinase domain and the Dk-helix in the CORB domain, is completely shielded from solvent, both ends are solvent exposed. The C-terminal end of the α3^ROC^ helix communicates with the C-terminus of the CORB Dk-helix and the active site cleft of the kinase domain while the N-terminal end is close to Switch II that is part of the G-protein sensor switch mechanism (Supplementary Figure S3).

### Switch II

In fl-LRRK2^INACT^ there is a hydrophobic ‘Knob' that is capped by hydrophobic residues in the α3^ROC^ helix (Figure 5). The residues in this knob are part of Switch II that was first defined in Ras (Supplementary Figure S3). H1405, H1407, and M1409 form the core of this ‘Knob' while W1434 and F1436 in the α3^ROC^ helix cap this knob. R1398 is part of Switch II motif and corresponds to Q61 in Ras which is one of two residues that is highly mutated in cancers. As seen in Supplementary Figure S3, the position of Q61 changes significantly depending on whether Ras is bound to GDP (top panel) or GTP (bottom panel). When Ras is in an inactive conformation, Q61 is solvent exposed and similar to the solvent exposed position of R1398 in inactive fl-LRRK2^INACT^ (Figure 5 and Supplementary Figure S3).

**Figure 6.**
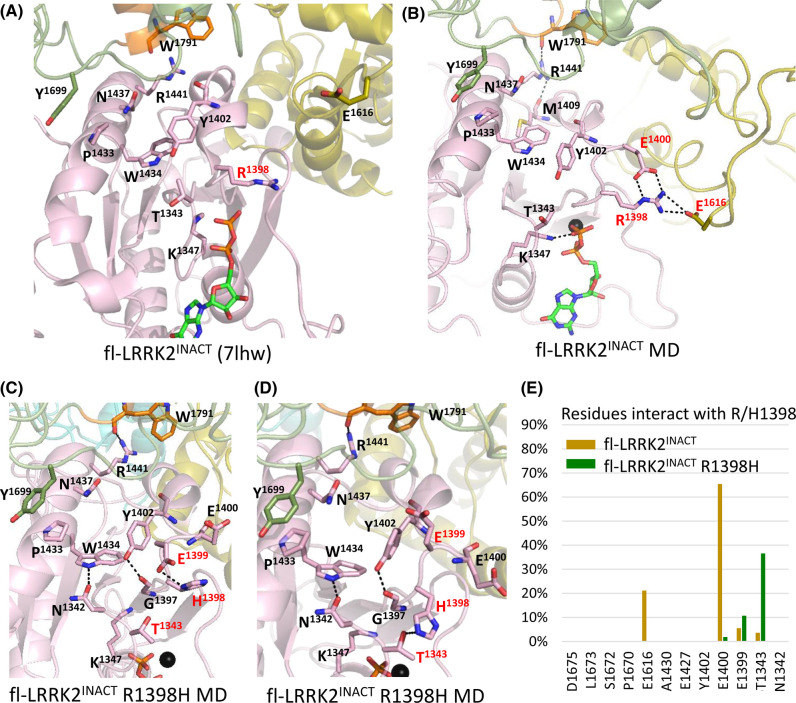
Simulations of R1398 in fl-LRRK2^INACT^ highlight interactions with E1616. (**a**) Cryo-EM structure of fl-LRRK2^INACT^ with highlighted key residues. (**b**) Representative GaMD simulation structure of fl-LRRK2 showing major interactions of R1398: R1398–E1400 and R1398–E1616. (**c**) Representative GaMD simulation structure of fl-LRRK2 R1398H mutant shows H1398 interacting with E1399 and pointing towards T1343. (**d**) Another representative structure shows the H1398 interacting with pT1343. (**e**) Table showing residue frequency of interactions with R1398 during simulation. For WT, major interactions are with E1400 and E1616, while for H1398, major interactions are with E1399 and T1343. For GaMD simulation trajectories see Supplementary Figure S4, which includes gif movies.

Our GaMD simulations capture some of the dynamic features of R1398 when it is in this inactive state (Figure 6 and Supplementary Figure S4). Although in the cryo-EM structure of inactive fl-LRRK2^INACT^ the side chain of R1398 is interacting primarily with E1400, an adjacent residue in the Switch II motif (Figure 6A), the simulations show that it also samples interactions with E1616 (Figure 6B,E), which lies in a more distal part of the CORA domain and is a dynamic region that comes close to the flexible linker that joins CORA and CORB (Figure 4). This region is much more flexible in fl-LRRK2^INACT^ than in LRRK2^RCKW^. This interaction can be quantitated more accurately in the bar graph (Figure 6E). We thus consider that this position of R1398, where it is solvent exposed and interacts with both E1400 and E1616, is a hallmark signature of inactive LRRK2.

R1398 is significant not only because of its homology to Q61 in Ras and its strategic position as part of Switch II but also because it is an unusual PD mutation site. Unlike all the other activating PD mutations, R1398H/K are *protective* mutations for both PD and IBD [18,19]. To explore the consequences of the Arg/His mutation on fl-LRRK2 we thus introduced *in silico* the R1398H mutation into our cryo-EM model of fl-LRRK2^INACT^ and carried out GaMD simulations. The results (Figure 6C–E and Supplementary Figure S4) show that H1398 is unable to reach to E1616 leading us to predict that it would be defective in its ‘off switch'. Can this explain why the kinase activity of fl-LRRK2 is activated by this mutation? Our kinetic data suggest weak activation of R1398H, which differs from the tightly regulated canonical activation mechanism, although R1398H is not as active as the G2019S mutant. To fully understand how fl-LRRK2 is locked into an inactive state, we need to delve more deeply into the LRR–ROC linker motif and its interactions with the ROC:CORA domain.

### Protein:Protein interfaces in fl-LRRK2^INACT^

**Figure 7.**
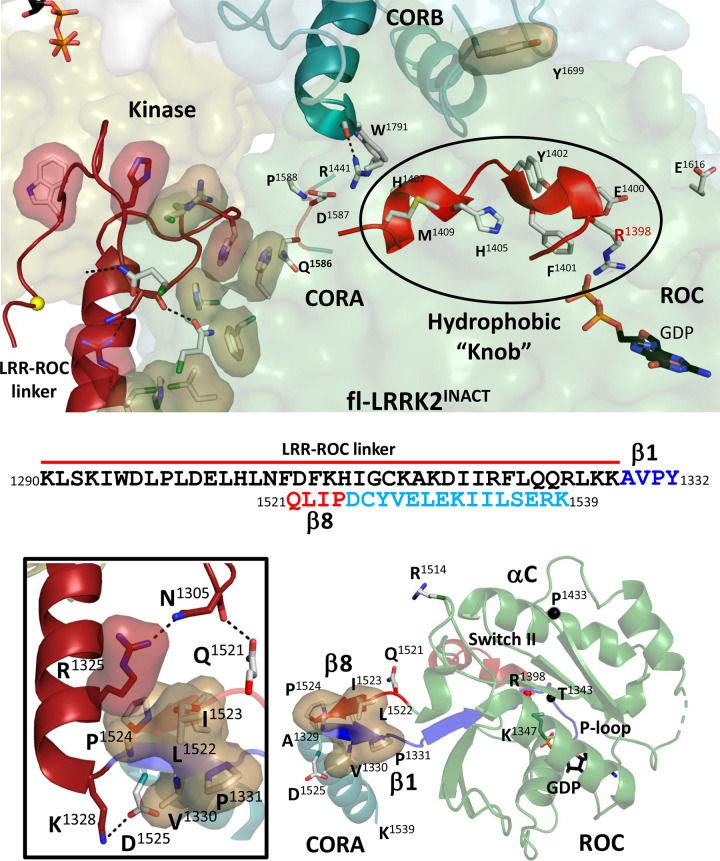
Conformation of the Switch II in the fl-LRRK2^INACT^. Top panel: In the fl-LRRK2^INACT^, the LRR–COR linker is ordered and packed against the ROC:CORA domains. In this structure, the Switch II residues form a hydrophobic knob (black circle). Middle panel shows the sequences of the Linker and β1/β8 strands of ROC domain. Bottom panel: Left shows the interactions of a key residue, R1325, with the β1/β8 strands of the ROC domain. Right shows how the β1/β8 strands are ordered. The Switch II and some key residues in the ROC domain are also shown. A more complete packing of the ROC:CORA domain is shown in Supplementary Figure S3.

We described previously a novel stable motif that links the LRR and ROC domains in the inactive conformation of fl-LRRK2. In our earlier description of the LRR–ROC linker we showed how the P + 1 loop of the kinase domain is occupied by W1295 in the stable linker motif [3]. We also showed how this linker motif is locked in a stable state by hydrophobic residues in the LRR domain and in the ROC:CORA domains. Here we focus on the hydrophobic interface between the linker and the ROC:CORA domains (Figure 7). In this structure, the Switch II motif is locked into a stable hydrophobic ‘Knob' that leaves R1398 solvent exposed in close proximity to E1616. The hydrophobic residues that interact with the linker are on the opposing surface of the ROC domain. In this structure, the ROC domain begins with a short β1 strand (residues 1328–1332) and ends with an antiparallel β8 strand (residues 1521–1525). Hydrophobic residues in these two β strands buttress up against R1325 in the LRR–ROC linker which was recently identified as another activating PD mutation (Figure 7) [20]. This linker motif is missing in LRRK2^RCKW^ and is disordered in active fl-LRRK2, so the hydrophobic interface is gone. This accounts for the dramatic increase in dynamics that was captured in Figure 4. Specifically, the regions corresponding to β1 and β8 are very stable in fl-LRRK2^INACT^ and highly dynamic in LRRK2^RCKW^. The ways that the LRR, ROC, and CORA domains each contribute to the hydrophobic packing and stabilization of the LRR–ROC linker is further emphasized in Supplementary Figure S5. Having now explored the structure and function of the kinase and GTPase domains in inactive fl-LRRK2^INACT^ we turn now to LRRK2^RCKW^.

### Conformation of the ROC:CORA domains in LRRK2^RCKW^

**Figure 8.**
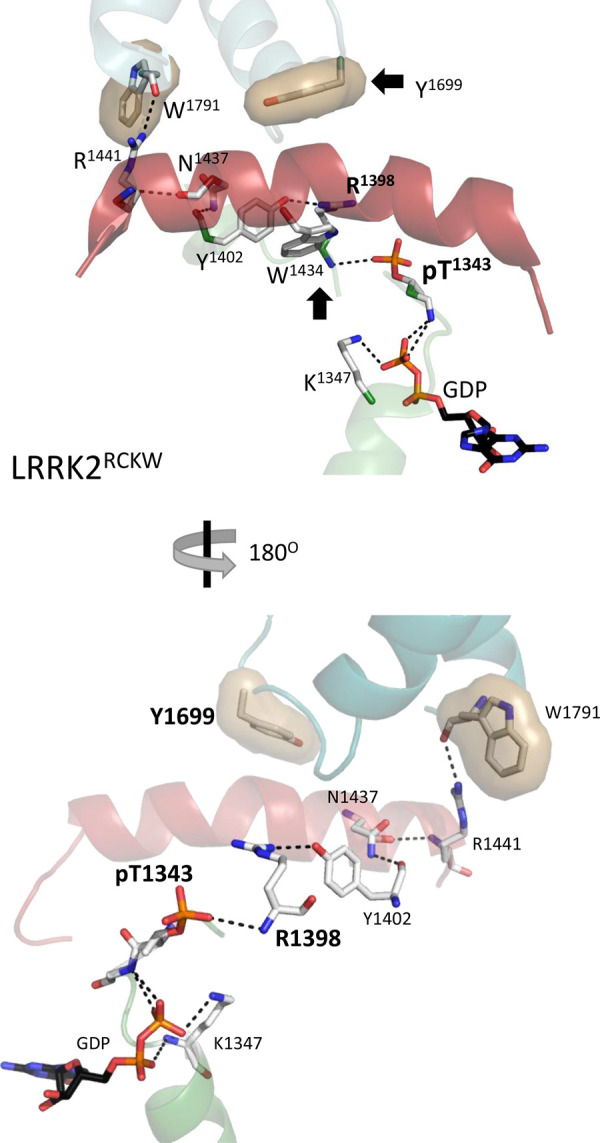
α3^ROC^ Helix in LRRK2^RCKW^. The key residues, R1398, Y1402, and Y1699 have a very different conformation in LRRK2^RCKW^ compared with fl-LRRK2^INACT^. R1398 has moved up and makes critical interactions with Y1402, which contributes to the repositioning of Y1699. pT1343 is also interacting with the backbone of R1398.

LRRK2^RCKW^ was originally defined as an inactive monomer although it had a tendency to form a trimer. When the protein was set up for cryo-EM studies, the buffer contained 0.5 mM GTP [8]. One of the advantages of cryo-EM is that one can capture different conformational states in the same structure, and the structure that was described had two unusual features that were not seen in the later structure of fl-LRRK2^INACT^. While both structures had bound GDP, LRRK2^RCKW^ also had trapped a phosphorylation site at T1343 (Figure 8). This residue is part of the P-loop that immediately precedes Switch I and is homologous to G12 in Ras (Supplementary Figure S3) which is also highly mutated in many cancers. Another striking feature in LRRK2^RCKW^ is that R1398 is rotated so that it is now at the ROC:CORB interface (Figure 8) whereas in fl-LRRK2^INACT^ R1398 is solvent exposed (Figure 5). As indicated above, R1398 is significant for several reasons as described above.

R1398 in LRRK2^RCKW^ makes critical interactions with residues in the α3^ROC^ helix, in particular, with Y1402, which contributes to the repositioning of Y1699. In fl-LRRK2^INACT^ the side chain of Y1699 is packed up against the side chain of P1433 in the α3^ROC^ helix while in LRRK2^RCKW^, and it is pointing towards the N-terminus of α3^ROC^ helix (Figure 5). In the CORB domain it is facing towards a hydrophobic cluster that includes W1791 and other hydrophobic residues in the Dk-helix of CORB. This position of Y1699 is described as a defining signature feature of active fl-LRRK2 [2]. We thus hypothesize that this LRRK2^RCKW^ structure has somehow trapped an ‘active-like' monomer that includes GDP and pT1343. We suggest, furthermore, that this position of R1398 is also a defining feature of active LRRK2.

**Figure 9.**
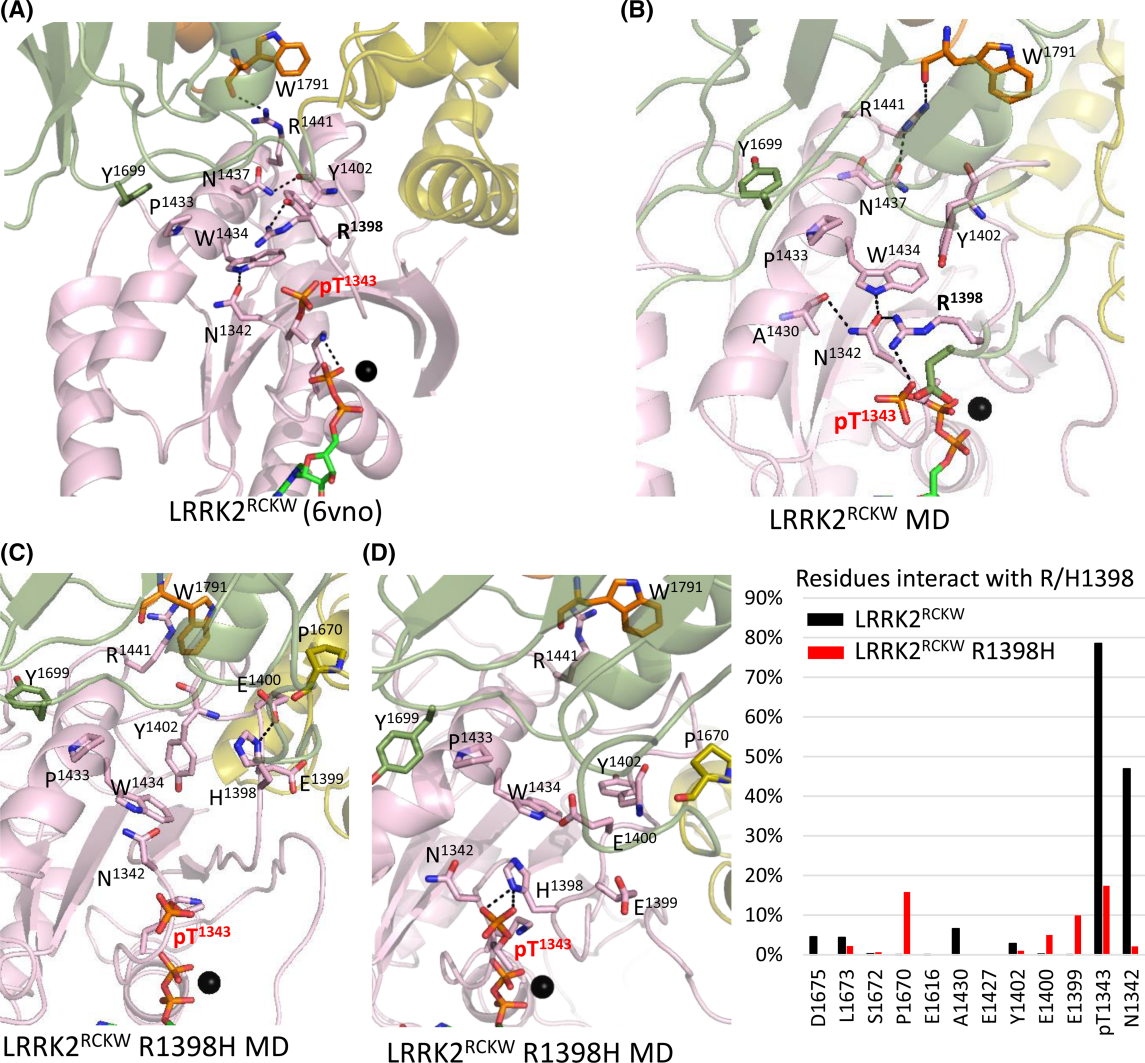
GaMD simulations showing R1398 going to the phosphate on Thr1343. (**a**) Cryo-EM structure of LRRK2^RCKW^ with highlighted key residues. (**b**) Representative GaMD simulation structure of LRRK2^RCKW^ showing major interactions of R1398: R1398–T1343 and R1398–N1342. (**c**) Representative GaMD simulation structure of LRRK2^RCKW^ R1398H mutant shows H1398 interacting with E1400 and pointing away from pT1343. (**d**) Another representative structure shows the H1398 interacting with pT1343. (**e**) Table showing residue frequency of interactions with R1398 during simulation. In WT, R1398 interacts mainly with pT1343 and N1342, whereas the mutant H1398 interacts less with pT1343 and more with P1670 and E1399. For GaMD simulation trajectories see Supplementary Figure S4, which includes gif movies.

In the cryo-EM structure, pT1343 is interacting with the backbone of R1398; however, the GaMD simulations indicate that the dominant interactions of the R1398 side chain are with the phosphate on T1343 (Figure 9 and Supplementary Figure S4). We then asked if these interactions are also seen when R1398 is mutated *in silico* to His. As seen in Figure 9C–E, the His greatly reduces its interaction with the phosphate; instead, it interacts with P1670. In the time frame of our simulations, however, it does not flip down to the solvent exposed position it assumes in fl-LRRK2^INACT^.

### Dynamic crosstalk between the kinase domain and the ROC:CORA domain is quenched in fl-LRRK2^INACT^ by the LRR–ROC linker

In our earlier study we showed how the LRR–ROC linker stabilized the Activation Segment of the kinase domain. Here we explore how the ROC domain is also stabilized by the linker. In particular, we focus on the β1–β8 motif defined earlier in Figure 4. This region has been largely ignored so far, but it is critical for several reasons (Figures 4 and 6). β1 strand (A1329) marks the beginning of the ROC domain while β8 is the end of the ROC domain and is fused directly to D1525, which is nominally the first residue in the CORA domain. In the full-length protein this β1–β8 motif is well-ordered and fused to the LRR–ROC linker (Figure 7) [3]. Residues from both the ROC and CORA domains, as well as from the LRR domain (Supplementary Figure S5), thus contribute to the hydrophobic shell that stabilizes the folded state of the LRR–ROC linker [3], and residues from the β1–β8 motif are part of this shell. When LRRK2 is activated by a Rab protein such as Rab29, the LRR–ROC linker becomes disordered leaving the exposed surface of the ROC domain free to interact with the exposed surface of the C-lobe of the kinase domain [2]. Like the LRR–ROC linker, the β1–β8 motif also undergoes an order/disorder transition in the LRRK2^RCKW^ construct. In the final LRRK2^RCKW^ structure the region corresponding to residues 1290-1328 was simply deleted because it was disordered [3]; the first detectable residue that defines the beginning of the ROC domain is A1329. However, this entire surface that was bound to the LRR–ROC linker is now highly dynamic as is captured by the GaMD simulations (Figure 4). But what happens to Switch II when the LRR–ROC linker is missing?

### Conformation of Switch II in LRRK2^RCKW^

**Figure 10.**
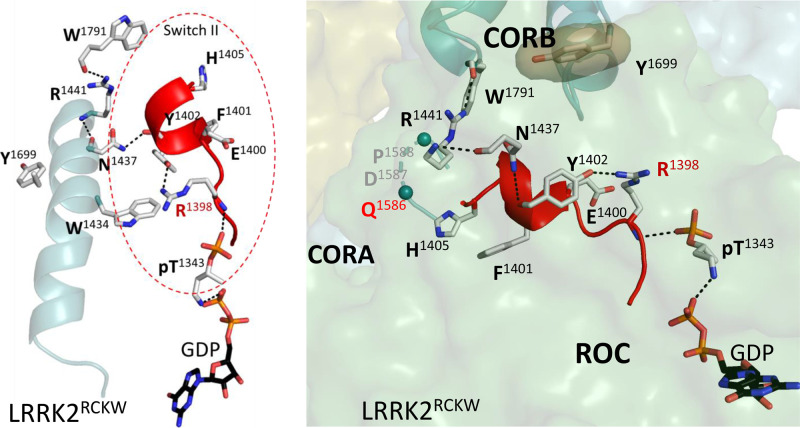
Conformation of the Switch II in the LRRK2^RCKW^. Left panel shows the α3^ROC^ helix and Switch II (red circle) with key residues highlighted. Right panel shows how this Switch II adopts a different conformation in LRRK2^RCKW^ compared with fl-LRRK2^INACT^ (Figure 7). The hydrophobic knob residues H1405 and F1401 in Switch II have rearranged significantly in LRRK2^RCKW^. They now point towards CORA and the region from residues 1406–1411 is disordered. Additionally, R1398 is recruited to the ROC:CORB interface.

In addition to the enhanced dynamics of the regions that are stable and bound to the LRR–ROC linker in fl-LRRK2^INACT^, there are changes in Switch II (Figure 10). For example, some residues in the hydrophobic knob defining Switch II (Figures 5 and 7) have significantly moved, and R1398 is recruited to the ROC:CORB interface, as described earlier. As seen in Figure 10, H1405 and F1401 that were part of the hydrophobic knob are now pointing towards the dynamic region that was created by the disordering of the LRR–ROC linker, and the region extending from residues 1406–1411 is disordered. H1405 interacts specifically with Q1586 in CORA, which is in close proximity to the Activation Segment in the kinase domain and is more stable in LRRK2^RCKW^ than in fl-LRRK2^INACT^. The GaMD simulations (Figure 4) also demonstrate these enhanced dynamics. Although further characterization in the ROC domain is required, our analyses suggests that this structure of LRRK2^RCKW^ represents a novel conformational state of Switch II.

### Dynamics of the kinase domain in fl-LRRK2^INACT^ versus LRRK2^RCKW^

**Figure 11.**
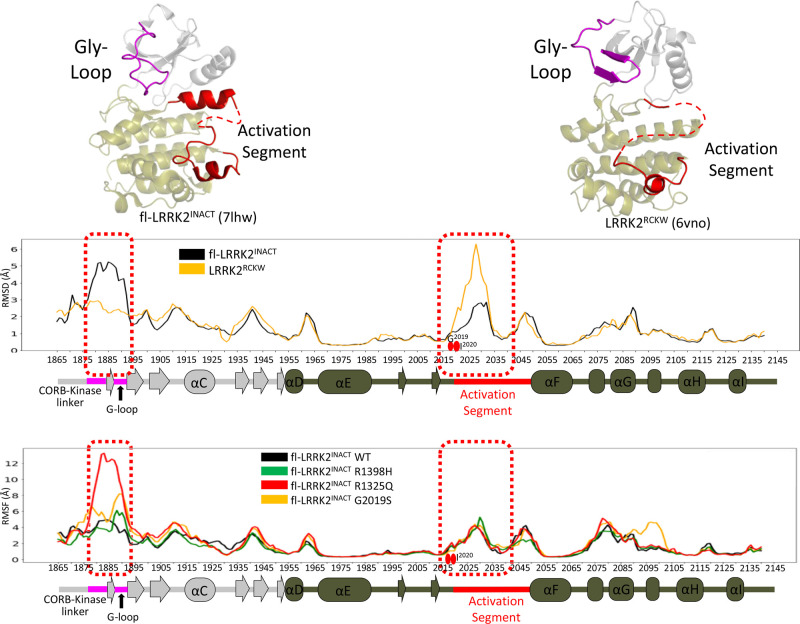
Dynamics of kinase domain in fl-LRRK2^INACT^ and LRRK2^RCKW^. The Cryo-EM structures of fl-LRRK2^INACT^ (top left) and LRRK2^RCKW^ (top right) highlight difference in the G-loop and part of the CORB-kinase linker (residue 1876–1893, colored in purple) and Activation Segment (residue 2019–2043, colored in red), and these differences are highlighted in the RMSF graphs. The kinase domain of fl-LRRK2^INACT^ and LRRK2^RCKW^ are compared in the middle panel. The G-loop and the linker to G-loop is more dynamic and has higher RMSF in fl-LRRK2^INACT^ compared with LRRK2^RCKW^, while the Activation segment has smaller RMSF in fl-LRRK2 than in LRRK2^RCKW^. Bottom panel shows the RMSF analysis of various PD mutations in the kinase domain of fl-LRRK2^INACT^. All of the mutants have a higher RMSF differences in the linker-to-G-loop region, with the R1325Q mutant having the highest RMSF. The G2019S mutant also shows higher RMSF in this region, whereas the R1398H mutant effects are limited to the G-loop.

While we have focused so far on the domain dynamics of the ROC domain, ultimately, we want to understand how and if mutations at the CORB interface and in the LRR–ROC linker influence the activity and the dynamics of the kinase domain. Our previous study showed not only how the LRR–ROC linker stabilized the C-Lobe of the kinase domain by docking of W1295 onto the P + 1 pocket of the Activation Segment and by an inhibitory helix that is formed by the DYG motif, but also showed that the G-Loop was highly dynamic [3]. Both of these features are captured perfectly in the GaMD simulations of the kinase domain in fl-LRRK2^INACT^ (Figure 11); the Activation Segment is very stable, and the G-Loop is highly dynamic. In our earlier study we compared the Activation Segment in fl-LRRK2^INACT^ and in LRRK2^RCKW^, and our findings are consistent with the simulations we show here. The Activation Segment in fl-LRRK2^INACT^ is very stable due in large part to the DYG inhibitor helix and the LRR–ROC linker while in LRRK2^RCKW^ it is disordered and very dynamic in the simulations [3]. The disorder of the Activation Segment in LRRK2^RCKW^ was also reflected in the HDX-MS data [12,13]. With HDX-MS we could then show protection of the Activation Segment with a type I inhibitor but enhanced exposure with a Type II inhibitor [21]. In Figure 11 we see that the G-Loop and part of the linker region connecting CORB and the region extending from the CORB domain over the N-Lobe of the kinase domain have the opposite properties. This region is more dynamic in fl-LRRK2^INACT^ and more stable in LRRK2^RCKW^. The C-terminal tail with phosphorylation sites at T2524 and S2525 also communicates with this space.

In Figure 11 bottom panel we show the effect of several PD mutations. G2019S mutation is located in the kinase domain and shows major effects on the G-loop, which is in the close proximity to the mutation site, and extension to the CORB:N-lobe linker region. This mutation also destabilizes the region around the G-helix. Surprisingly, the effects of the R1398H mutation in the ROC domain can also be sensed in the kinase domain. These effects are more subtle and more localized to the G-loop but nevertheless significant.

### Importance of the R1325Q PD mutation in the LRR–ROC linker

Most of the well-characterized PD mutations in LRRK2 lie either at the DYG motif, which defines the interface between the N- and C-Lobes of the kinase domain or in the ROC:CORB interface. In contrast, R1325 is in the LRR–ROC linker that contributes to the ROC-COR-A interface. This interface, unlike the other two, undergoes an order–disorder transition upon activation. It is a well-formed hydrophobic interface in fl-LRRK2^INACT^ due in large part to the β1–β8 motif in the ROC domain and the interaction with N1305. which is also in the linker (Figure 7). The interface with the methylene groups of R1325 is achieved primarily by hydrophobic residues in β8 (L1522, I1523, and P1524) while N1305 interacts with Q1521 which is the first residue of β8. It is thus reasonable to predict that this mutation would break or weaken the interface and in part mimic physiological activation that is mediated by Rab29 binding. GaMD simulations showed how removal of NtDs including the LRR–ROC linker leads to the global conformational changes in LRRK2^RCKW^ (Figure 4). Using GaMD simulations of fl-LRRK2^INACT^ we also asked *in silico* how this mutation influences the ROC:CORA interface and also if it influences the dynamics of the kinase domain. Most striking is the dramatic effect that this mutation has on the extended G-Loop region of the kinase domain (Figure 11), which is toggling between an inactive nucleotide free state and a nucleotide bound state in fl-LRRK2^INACT^. It becomes highly dynamic in response to the mutation which potentially primes it for activation.

## Discussion

With LRRK2 we have an extraordinary opportunity to delve deeply into the structure, function, and allosteric regulation of a large multi-domain kinase that has enormous biological relevance. Because the kinase domain of LRRK2 does not express as a stable protein, it was necessary to focus on the larger multi-domain constructs. In retrospect, this was quite fortunate as we would otherwise have spent much time and energy on analyzing just the kinase domain, which is what is done for most other kinases such as BRaf and the EGF receptor. Only recently with structures of full-length BRaf bound to 14-3-3 proteins, that can alternatively stabilize active and inactive conformations, can we fully appreciate why the kinase domain alone gives only a partial and incomplete picture [22]. The fact that high-resolution cryo-EM structures of the CtDs of LRRK2 followed by cryo-EM structures of full-length LRRK2 in its active and inactive states is truly unprecedented. It allows us to move across many scales to understand protein dynamics and allostery and to look in new ways at distal allosteric sites that have the potential to be therapeutic targets. We have previously used HDX-MS to map solvent exposed and solvent shielded regions of LRRK2^RCKW^, which is not only quite large but also contains both a kinase domain and a GTPase domain. LRRK2 also is highly unusual in the kinase community where kinases and GTPases communicate often in signaling cascades but are not both part of the same polypeptide chain. In addition to mapping solvent accessibility with HDX-MS, we can use computational tools to interrogate the dynamic features of LRRK2, and here we can use both full-length LRRK2 and LRRK2^RCKW^ and compare them. We begin with a comparative analysis of the catalytic activities, both kinase and GTPase, and then use GaMD simulations to create a dynamic portrait of these two static structures.

Before exploring the potential crosstalk that takes place between the kinase domain and the GTPase domain we needed to define more rigorously the motifs of the ROC:CORA domain and show how they differ in the two structures. In addition to Switch I and Switch II, which are canonical features of all GTPases, we initially concentrated on the α3^ROC^ helix of the ROC domain which is very hydrophobic and forms the surface that communicates with the CORB domain (Figures 2 and 5). The importance of this surface is highlighted by the fact that many PD mutations lie at this interface. A defining feature of active fl-LRRK2 is the position of Y1699, which is one of the PD activating mutations. In active fl-LRRK2 Y1699 is facing toward W1791 at the C-terminus of the CORB Dk-helix described earlier (Supplementary Figure S1) [2]. Adjacent to W1791 are two additional PD mutation sites, R1441 and N1437, which lie at the C-terminus of the α3^ROC^ helix. R1441 and W1791 both are also close to the Activation Segment of the kinase domain. In the structure of LRRK2^RCKW^ that was first captured by cryo-EM there is something unusual (pdb: 6vno). Specifically, in this structure, which had GTP in the buffer prior to data collection, there is a bound GDP as there is in fl-LRRK2^INACT^, but in LRRK2^RCKW^ T1343 is also phosphorylated. This P-site was reported previously [23,24], but its potential functional significance or physiological relevance was not discussed. This residue is potentially important because it is homologous to G12 in Ras, a residue that is mutated in many tumors. Another unusual residue is located in this region, R1398; it is interesting and potentially important because it is the only mutation described so far that is protective against PD and it is also protective for IBD. In LRRK2^RCKW^ this residue is also rotated. In fl-LRRK2^INACT^ it is solvent exposed while in LRRK2^RCKW^ it is rotated up and is at the ROC:CORB interface. R1398 is also potentially biologically important because it is homologous to Q61 in Ras which is the second residue that is commonly mutated in many cancers. Furthermore, it is part of Switch II, which undergoes significant changes in LRRK2^RCKW^ compared with fl-LRRK2^INACT^. Since the position of Q61 in Ras changes significantly upon activation (Supplementary Figure S2). we predict that these changes in the position of R1398 may also be a hallmark signature of active LRRK2.

Given the comparisons with Ras and other GTPases, which highlight the importance of Switch II, we hypothesize that the LRRK2^RCKW^ structure has trapped a putative GTP binding state, where the γ-phosphate has been most likely transferred to T1343. We predict that the sidechain of R1398 is recruited to the ROC:CORB interface by the γ-phosphate of GTP, which then gets hydrolyzed. Whether pT1343 is an intermediate in hydrolysis remains to be determined, but it is certainly a possibility. It is not clear why this state was transiently trapped in this structure, but it is not seen so far in the other structures. Was the phosphorylated state of LRRK2 specifically enhanced by the trimer used for symmetry averaging in the original structure determination? In solution would this phosphate be hydrolyzed by formation of CORB:CORB dimer which is thought to be the next step in LRRK2 activation? In all of the structures so far, the guanine ring is not capped by the α5 helix, which is a characteristic feature of GTPase activation. This surface is solvent exposed in both LRRK2 structures. It remains to be determined if this surface is modified by microtubule binding or if it interacts with another protein. These are questions that need to be resolved in future studies.

Finally, we explore the role of the folded and stable LRR–ROC linker that we described earlier in fl-LRRK2^INACT^ [3]. This small motif, which toggles between an ordered and disordered state, plays a critical role in LRRK2 activation. In inactive LRRK2 where the LRR domain is wrapped around the kinase domain locking it into an inhibited state, the motif is very stable due in large part to a hydrophobic shell that is formed by residues from the LRR-domain and from the ROC:CORA domains. The stability of this motif was confirmed by MD simulations [3]. Another activating PD mutation was recently described in the LRR-ROC linker, R1325Q [20]. Here we show how R1325 is stabilized in fl-LRRK2^INACT^ by beta strands 1 and 8 which begin and end the ROC domain (Figures 4, 7 and Supplementary Figure S4). In LRRK2^RCKW^ the LRR–ROC linker is missing and the region corresponding to and surrounding beta strands 1 and 8 is highly disordered (Figure 4).

To ask if mutations influence the dynamic features of the kinase domain in LRRK2, we compared the GaMD simulations of fl-LRRK2^INACT^ after introducing three mutations *in silico*, and the consequences are profound (Figure 11). We first explored the effects of the highly activated G2019S mutation, which is in the kinase domain and part of the critical interface between the N- and C-Lobes. As seen in Figure 11, this mutation influences the G-Loop region as well as the region surrounding the G-helix which is a canonical docking site for kinase substrates [25]. We next asked if the R1398H mutation in the ROC domain could be sensed by the kinase domain. The effect is not as pronounced as the G2019S mutation but nevertheless significant. Localized residues in the G-Loop and the Activation Segment become more dynamic compared with fl-LRRK2^INACT^. Thus, based on our GaMD simulations, both mutants appear to influence the dynamic features of the kinase domain.

Finally, we show, using *in silico* mutagenesis of fl-LRRK2^INACT^, that mutation of R1325 also has a profound effect on the G-Loop and the N-Lobe of the kinase domain. This entire region, which we referred to earlier as the N-lobe capping region, is especially important because it is where the C-terminal tail of LRRK2 lies, and there are potential interactions with the linker extending from CORB to the kinase domain [3]. In addition, at least one phosphorylation site, T2524, lies in this region. This region appears to be acutely sensitive to the R1325Q mutation, and our results suggests that there is important crosstalk taking place between the linker and its adjacent domains even when the linker is disordered following the activation of LRRK2.

## Methods

### Transfection of HEK293-T cells

Expression of each Flag-Strep-Strep-tagged (FSS) LRRK2 construct (fl-LRRK2 and LRRK2^RCKW^) was proceeded in HEK293-T cells (human embryonic kidney cells carrying the temp sensitive mutant of the SV-40 large T-antigen, DSMZ, DSMZ-No:ACC635). For transfection, 1.0 × 10^7^ HEK293T cells were seeded in each of ten 15 cm Ø cell culture dishes and incubated for 24 h at 37°C and 6% CO2 in Dulbecco's Modified Eagle Medium (DMEM) high glucose (w. l-Glutamine; w.o. Sodium Pyruvate, biowest) supplemented with 10% fetal bovine serum (FBS). The cells were transfected by adding a mixture of 15 µg plasmid DNA (pcDNA 3.0-FSS-LRRK2 [aa1–2527] and pcDNA 3.0-FSS-LRRK2^RCKW^ [aa1327–2527]), 150 µl PEI (Polyethylenimine) (1 µg/µl) and 1.5 ml DMEM high glucose, that was preincubated for 30 min, per dish. After 24 h, medium was exchanged with fresh DMEM (high glucose, +10% FBS). Cells were harvested after further 24 h and pellets were stored at −20°C.

### Purification of overexpressed FSS-LRRK2 constructs from HEK293-T cells

Pellets were resuspended in 10 ml of ice-cold lysis buffer (25 mM Tris–HCl pH 7.5, 150 mM NaCl, 10 mM MgCl_2_, 0.5% Tween 20, 500 µM GDP, cOmplete™ EDTA free protease inhibitor cocktail [Roche], PhosSTOP™ [Roche]) and incubated for 30 min at 4°C on a rotating wheel to lyse cells. Following centrifugation at 42 000×***g*** and 4°C for 40 min and a filtration step (0.45 µm sterile filter) to remove cell debris, the supernatant was loaded onto a Streptactin Superflow column (0.5 ml bed volume, IBA Goettingen) and purification was performed according to the manufacturer's protocol while all buffers were supplemented with 500 µM GDP (Biolog Life Science Institute) and 10 mM MgCl_2_. The purified LRRK2 constructs were stored at −80°C containing 10% Glycerol and 0.5 mM TCEP. The protein concentrations were determined according to Bradford (1976).

### Microfluidic mobility shift assay to quantify kinase activity

Kinase activities were quantified with Microfluidic mobility shift assay (MMSA) using 1 mM ATP and 1 mM LRRKtide (RLGRDKYKTLRQIRQ-amide, GeneCust) as substrates. Two stock solutions were prepared, each containing 2× concentration: substrate solution (1900 µM LRRKtide, 100 µM Fluorescein-LRRKtide, 2 mM ATP) and enzyme solution (100 nM LRRK2 [functional protein concentration], 20 mM MgCl2, 1 mM GDP). Both stock solutions were prepared using standard kinase buffer (25 mM Tris–HCl, pH 7.5, 50 mM NaCl, 0.1 mg/ml BSA, 1 mM DTT). Reactions were started by mixing both solutions in a 1 : 1 ratio in a 384 well-plate and measurements were performed at 30°C. Substrate conversions were monitored for 60–90 min using a LabChip EZ Reader (PerkinElmer). The slope (conversion rate, [m] = %/min) of the percental conversion plotted against the time was determined using a linear fit model of GraphPad Prism 6 and was converted into reaction velocity ([v0] = µmol/min) and further into observed turnover numbers k_obs_ [min^−1^]. Experiments were performed at least in duplicates of duplicates for two independent LRRK2 expressions. To determine significant differences between LRRK2 wt and mutant activity a two-sided unpaired *t*-test (parametric) with a confidence interval of 95% was performed.

### Titration assays to determine functional protein concentration

Titration assays were performed to determine the functional protein concentrations of the LRRK2 variants using the high-affinity inhibitor MLi-2 (Merck, U.S.A.). Therefore, 24 µl of Buffer I (25 mM Tris–HCl, 50 mM NaCl, 20 mM MgCl_2_, 1 mM GDP, 1 mM DTT, 0.5 mg/ml BSA, 52.1/104.2 nM LRRK2 [total protein concentration after Bradford were mixed with 1 µl of a MLi-2 dilution series (50× concentrated) prepared in 100% DMSO [26]. The reactions were started by mixing 10 µl of this reaction mix with 10 µl of Buffer II (25 mM Tris–HCl, 50 mM NaCl, 1 mM DTT, 0.5 mg/ml BSA, 1900 µM LRRKtide, 100 µM Fluorescein-LRRKtide, 300 µM ATP) in a 384 well-plate and monitored for 60–90 min with a LabChip EZ Reader (PerkinElmer). The resulting conversion rates were plotted against the respective MLi-2 concentrations and the *x*-axes intersection of the respective linear fit was determined to obtain the functional protein concentrations using GraphPad Prism 6 (assuming a 1 : 1 binding of MLi-2).

### Radioactive charcoal assay to determine GTPase activity

GTPase activity was determined by quantifying GTP hydrolysis rates using a radioactive approach after Higashijima et al. [27], that was adapted and optimized to allow detection of low GTP turnover rates. [γP^32^]-GTP was used as radioactive labled substrate. P^32^ decays in a β-decay which can be detected as Cherenkov radioation. GTPase standard conditions were chosen for the stock solution (50 mM Tris–HCl, pH 7.5, 150 mM NaCl, 10 mM MgCl2, 1 mM DTT, 0.1 mg/ml BSA, 5% glycerol). The reaction was started by mixing 40 µl 2× GTP-mix (final 3 mM GTP, 25 nM [γP^32^]-GTP) with 40 µl 2× LRRK2 dilution (final 200 nM) and incubated at 30°C. Immediately after mixing the LRRK2 dilution with the GTP-mix, 10 µl sample (0’, background signal) were transferred into 300 µl charcoal solution (10% activated charcoal (w/v) in 20 mM H_3_PO_4_) and mixed well. The charcoal was previously saturated with phosphoric acid, thus not able to bind free phosphates which enables separation. Further samples were taken after 5, 10, 15, 20 and 25 min and treated analogously. One additional sample was transferred in ddH_2_O to serve as a total signal reference. Subsequently, the sample-containing charcoal solutions were transferred into DNA binding columns (Omega) that were placed in fresh 1.5 ml reaction tubes followed by 2 min centrifugation at 4000×***g*** to remove the charcoal. The columns were discarded and 150 µl of the diluted samples were transferred into scintillation tubes, filled with 5 ml ddH_2_O, allowing emission of Cherenkov radiation. Detection was achieved by the Hidex 300 SL liquid scintillation counter employing three photomultiplier tubes (PMT). Counting was proceeded for 80 s with a maximum limit of 30.000.000 counts, ionized delay of 5 s and a coincidence time of 35 ns. To determine time resolved substrate conversion, the total signal was first corrected by subtraction of the background signal (Netto Bq_total_ = Bq_total_ − Bqt = 0) and then used to calculate percentage conversion

$$(\mathrm{conversion}\;[\text{\%}]=\frac{Bq_{t=x}}{\mathrm{Netto}\;Bq_{\mathrm{total}}}\times 100)$$

which is used to calculate the amount of converted GTP by multiplying it by the amount GTP used in the assay

$$(\mathrm{converted}\;\mathrm{GTP}\;\left[\mu \mathrm{mol}]=\mathrm{GTP}[\mu \mathrm{mol}\right]\times \frac{\mathrm{conversion}\;\left[\text{\%}\right]}{100}).$$

The linear fit's slope of the time dependent GTP conversion equals the conversion rate [µmol/min] and can be converted into observed turnover number k_obs_ [min^−1^]. To determine significant differences between LRRK2 wt and mutant activity a two-sided unpaired *t*-test (parametric) with a confidence interval of 95% was performed.

### Gaussian accelerated molecular dynamics (GaMD) simulation

The LRRK2^RCKW^ and fl-LRRK2 simulation models were prepared using cryo-EM structures (pdb: 6vp6 and 7lhw). The missing loops in the protein structure were modeled using Modeller [28], while the missing sidechains and charge states of ionizable residues at neutral pH were built using the Protein Preparation Wizard. Hydrogens and counter ions were added, and the resulting models were solvated in a cubic box of TIP4P-EW water molecules and 150 mM KCl with a 10 Å buffer in AMBER tools [29]. Energy minimization, heating, and equilibration steps were performed using AMBER16, with the CPU code used for minimization and heating and the GPU code used for equilibration. Parameters from the Bryce AMBER parameter database were used for phosphoserine and phosphothreonine [30]. The systems were minimized through various steps, including hydrogen-only minimization, solvent minimization, ligand minimization, side-chain minimization, and all-atom minimization. The systems were heated from 0 K to 300 K over 200 ps with 2 fs time-steps and 10.0 kcal/mol/Å position restraints on protein, with temperature maintained by the Langevin thermostat. Constant pressure equilibration with an 8 Å non-bonded cut-off and particle mesh Ewald was performed for 300 ps with protein and peptide restraints, followed by 900 ps of unrestrained equilibration. Gaussian accelerated MD (GaMD) was used on GPU-enabled AMBER16 to enhance conformational sampling [6]. GaMD applies a Gaussian distributed boost energy to the potential energy surface to accelerate transitions between meta-stable states while allowing accurate reweighting. Both dihedral and total potential boosts were used simultaneously. Potential statistics were collected for 2 ns followed by 2 ns of GaMD during which boost parameters were updated. Each GaMD simulation was equilibrated for 10 ns. Finally, for each construct, three independent replicates of 210 ns of GaMD simulation were used for analyses.

## Data Availability

The findings of this study are supported by the data within the article and its supplementary materials. The models of LRRK2 MD simulations can be accessed at https://doi.org/10.6084/m9.figshare.22593448, and additional information can be obtained by contacting the corresponding author, S.S.T.

## Open Access

Open access for this article was enabled by the participation of University of California in an all-inclusive *Read & Publish* agreement with Portland Press and the Biochemical Society under a transformative agreement with UC.

## Abbreviations

DMEM: Dulbecco's Modified Eagle Medium
FBS: fetal bovine serum
FSS: Flag-Strep-Strep-tagged
GaMD: Gaussian Accelerated Molecular Dynamics
HDX-MS: Hydrogen-Deuterium exchange Mass Spectrometry
IBD: inflammatory bowel disease
LRRK2: LRR-kinase 2
PD: Parkinson's Disease
ROC: Ras-of-complex
