# Supplementary material for: Capturing the domain crosstalk in full length LRRK2 and LRRK2RCKW
Source: Biochem J. 2023 Jun 12;480(11):815–33. doi: 10.1042/BCJ20230126 (PMC10317166; doi:10.1042/BCJ20230126)
Supplement: Supplementary Material [file BCJ-480-815-s1.pdf]

Figure S1

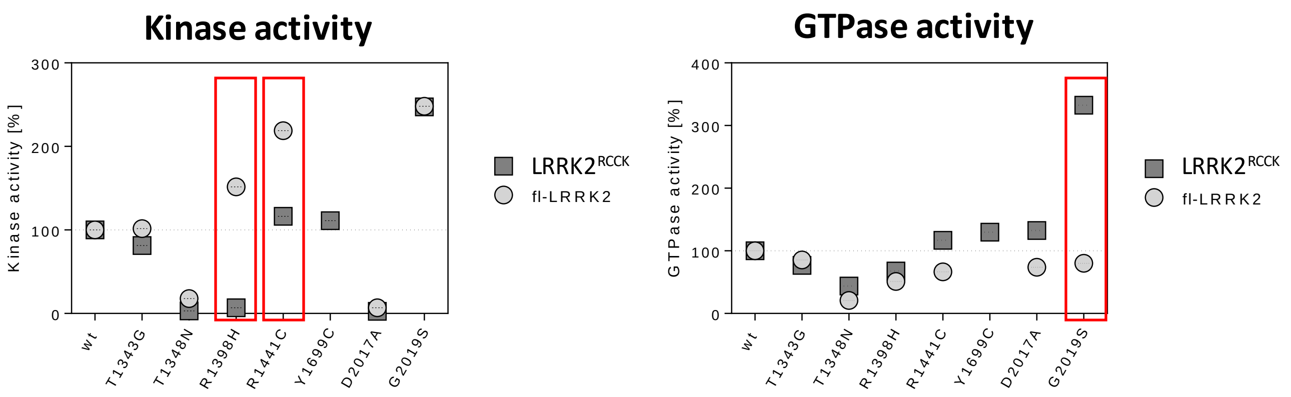

Percentage activity [%]

Kinase activity	fl-LRRK2	LRRK2^{RCKW}
wt	100	100
T1343G	101.6	81.4
T1348N	17.8	3.0
R1398H	151.7	6.8
R1441C	218.9	116.3
Y1699C	n.d.	111.1
D2017A	6.9	2.6
G2019S	248.2	247.4

GTPase activity	fl-LRRK2	LRRK2^{RCKW}
wt	100	100
T1343G	85.5	76.7
T1348N	20.7	43.9
R1398H	51.2	68.0
R1441C	66.5	116.7
Y1699C	n.d.	129.8
D2017A	73.7	132.4
G2019S	80.2	332.5

Absolute activity [min^{-1}]

Kinase activity	fl-LRRK2	LRRK2^{RCKW}
wt	68.9 ± 4.8	100.8 ± 5.7
T1343G	69.7 ± 11.6	82.1 ± 7.1
T1348N	12.1 ± 1.1	3.1 ± 1.7
R1398H	106.0 ± 1.3	6.9 ± 4.5
R1441C	200.3 ± 19.2	147.4 ± 26.6
Y1699C	n.d.	149.7 ± 18.3
D2017A	4.8 ± 2.7	2.6 ± 0.9
G2019S	168.3 ± 14.8	249.5 ± 5.0

GTPase activity	fl-LRRK2	LRRK2^{RCKW}
wt	2.1 ± 1.2	0.8 ± 0.5
T1343G	2.1 ± 0.7	0.8 ± 0.5
T1348N	0.5 ± 0.04	0.6 ± 0.4
R1398H	1.3 ± 0.4	0.7 ± 0.4
R1441C	1.7 ± 0.3	0.8 ± 0.1
Y1699C	n.d.	0.9 ± 0.01
D2017A	1.8 ± 0.2	0.9 ± 0.2
G2019S	2.0 ± 0.5	2.3 ± 0.7

Figure S1. Summarized enzymatic activities of all tested LRRK2 variants. Side-by-side comparisons of the percentage activities of fl-LRRK2 and LRRK2^{RCKW} variants (top panel) show that the most apparent differences are contributed by the PD related mutations R1398H, R1441C and G2019S (red rectangles). The mid panel shows relative activities [%] and the lower panel shows the absolute activities [min-1] (± SD) of all tested LRRK2 variants (n.d.: not determined).

Figure S2

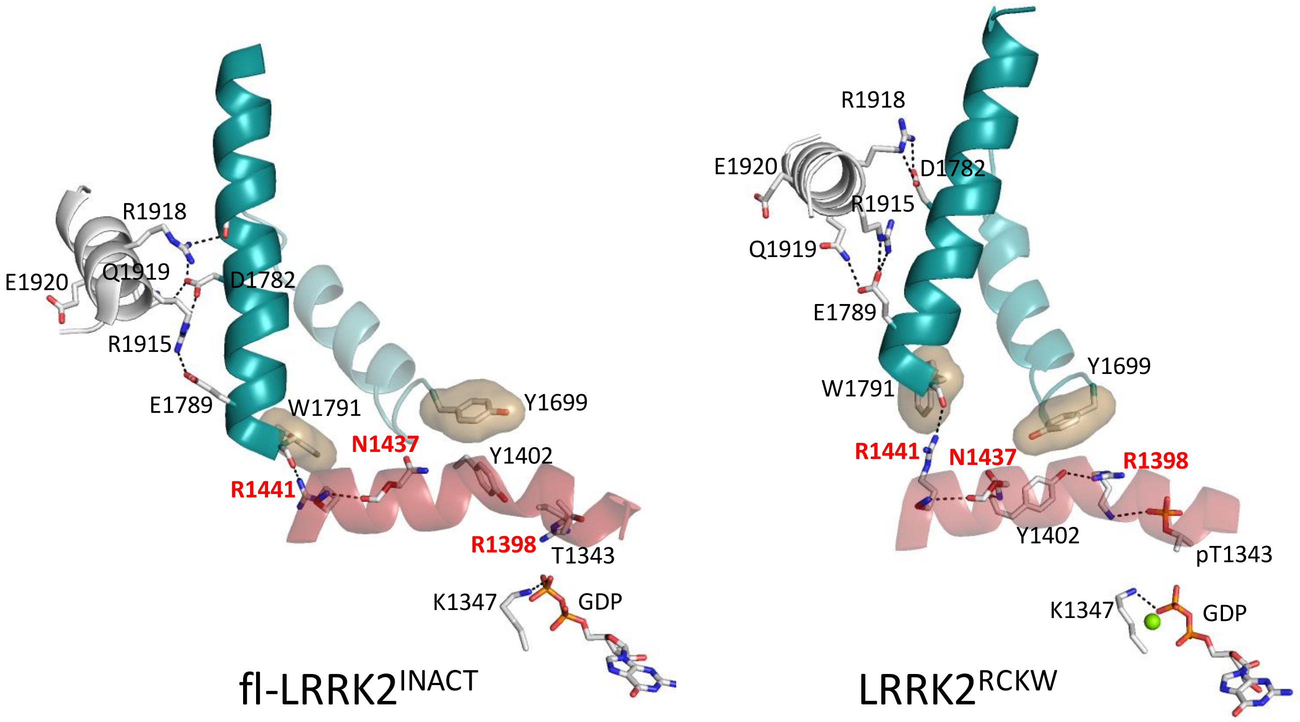

Figure S2. Rigid body motion of key helices in fl-LRRK2^{INACT} and LRRK2^{RCKW}. Here we show how the two helices in the COR-B domain interact with αC helix in the kinase domain and with the interface with the a3^{ROC} helix in the ROC domain. PD mutations at the ROC:CORB interface are highlighted in red.

Figure S3

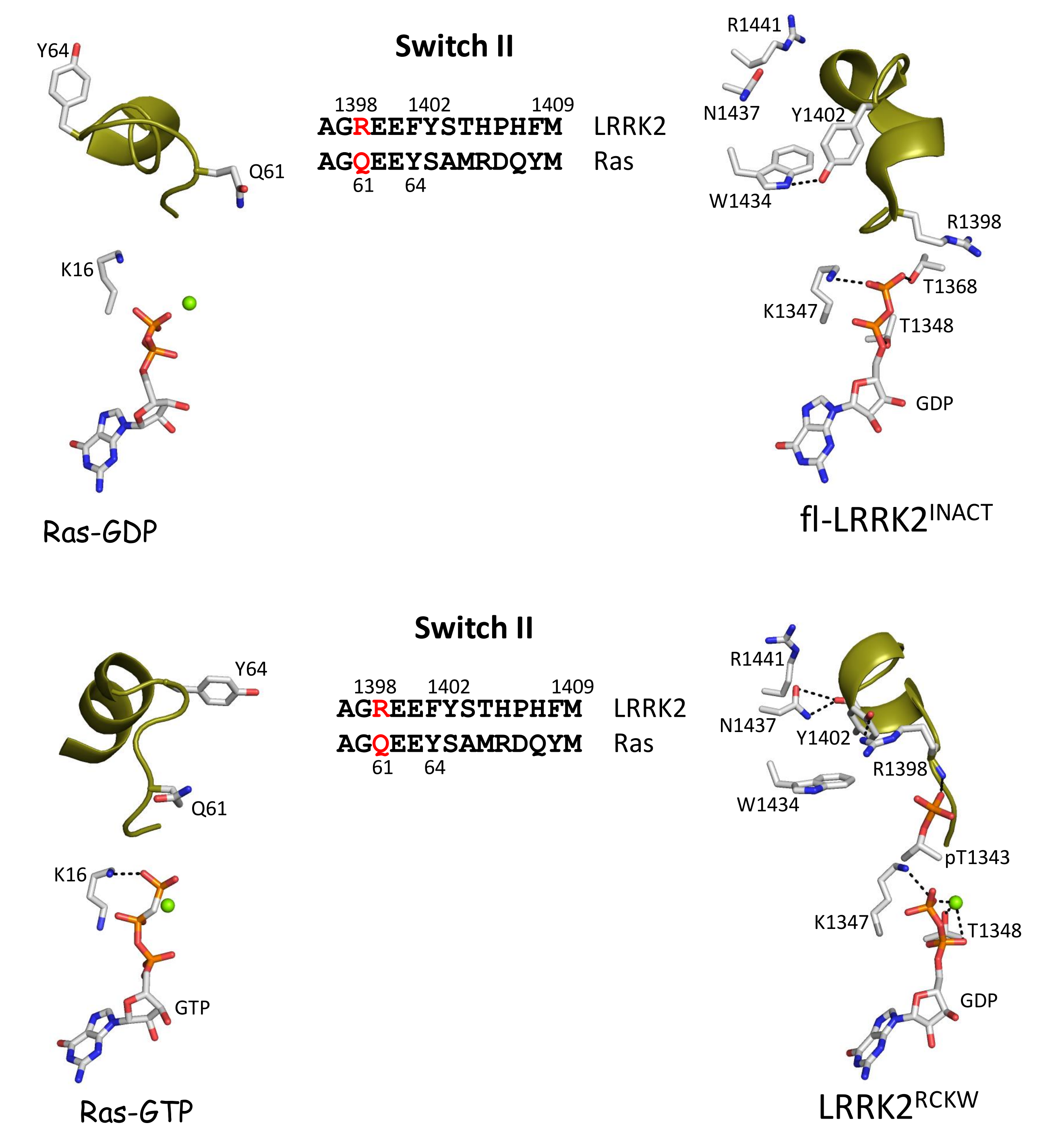

Figure S3. Comparing conformations of Switch II in GDP/GTP bound Ras with conformations of Switch II in fl-LRRK2^{INACT} and LRRK2^{RCKW}. Top panel compares the GDP-bound state of Ras with fl-LRRK2$_{INACT}$. Bottom panel captures the major conformational changes in Ras when GTP is bound, and compares them to the changes we see in Switch II in LRRK2^{RCKW}. The Switch II sequences of Ras and LRRK2 are shown in the Middle.

Figure S4

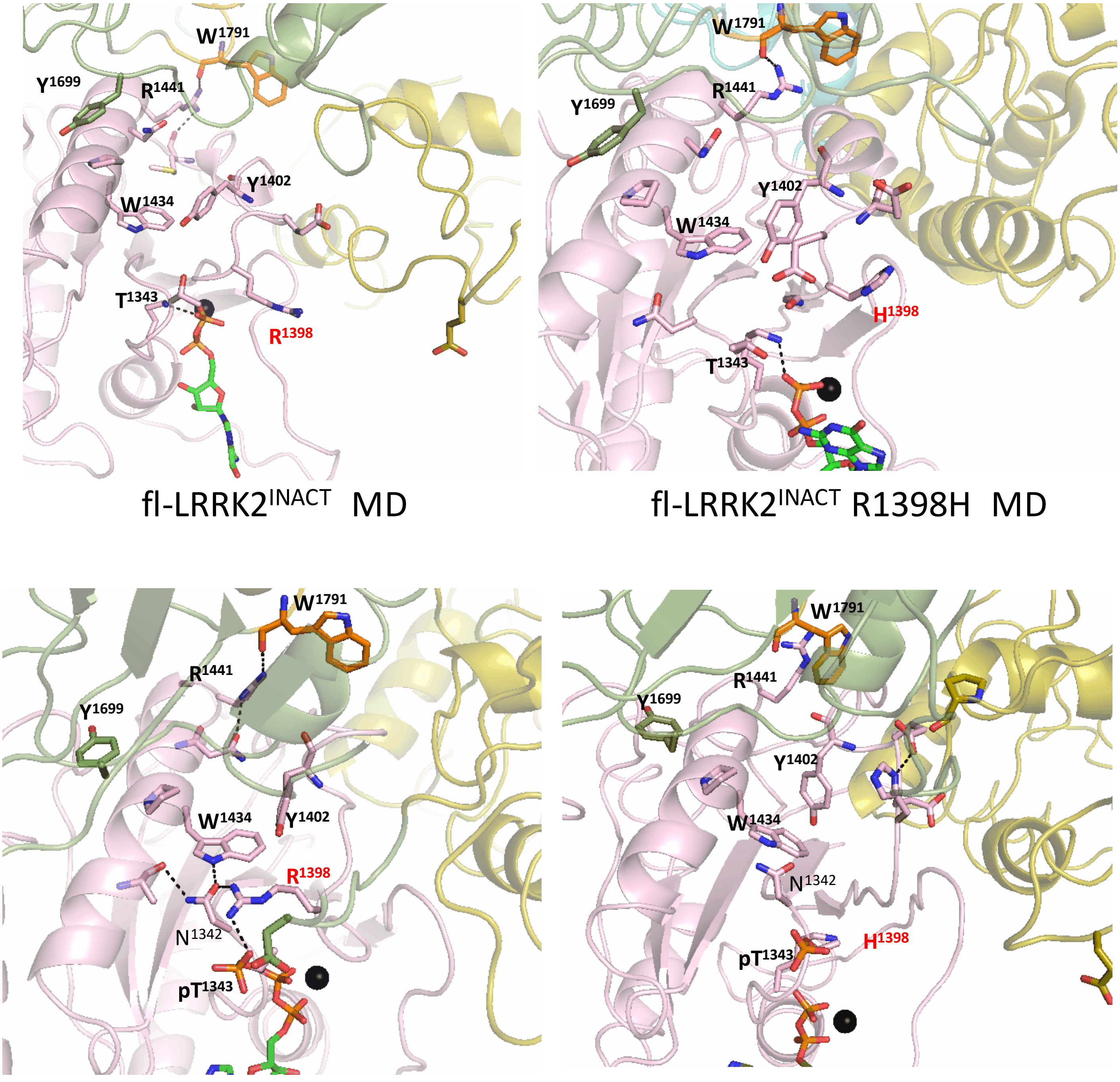

Figure S4. GaMD simulations of the ROC domain in fl-LRRK2^{INACT} and LRRK2^{RCKW}. Top panel shows fl-LRRK2^{INACT}; bottom panels show LRRK2^{RCKW}. Left panels show WT LRRK2, while right panels show R1398H mutations.

Figure S5

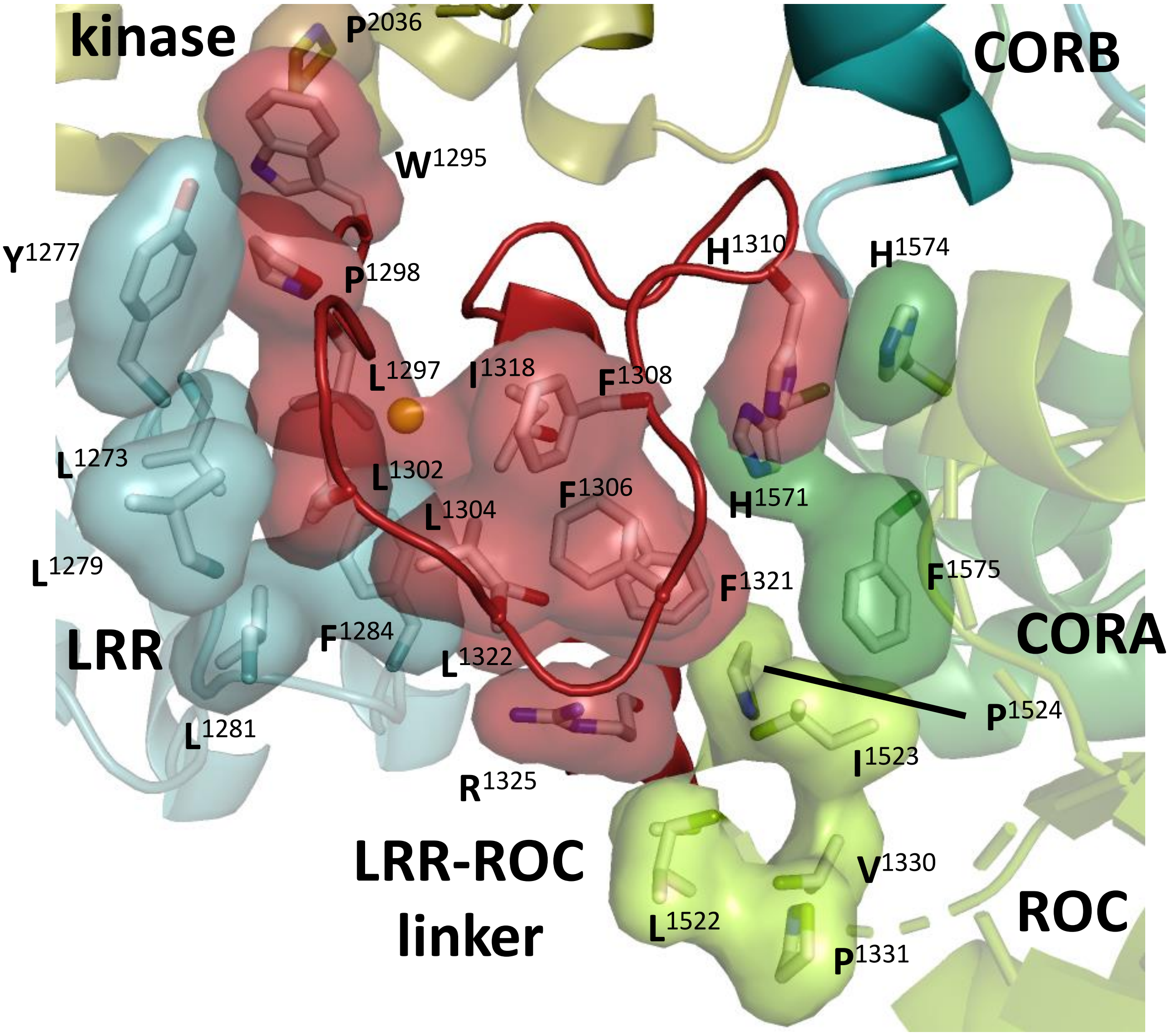

1290**KLSKIWDLPLDELHLNFDFKHIGCKAKDIIRFLQQRLKK**1328

fl-LRRK2^{INACT}

Figure S5. Hydrophobic interface of LRR-ROC linker. The LRR-ROC linker in fl- LRRK2^{INACT} is ordered by hydrophobic packing from residues in the LRR (teal), ROC (lime), and COR-A (dark green) domains. The C-lobe of the kinase domain is in tan.
